# Recently Discovered Interstitial Cell Population of Telocytes: Distinguishing Facts from Fiction Regarding Their Role in the Pathogenesis of Diverse Diseases Called “Telocytopathies”

**DOI:** 10.3390/medicina55020056

**Published:** 2019-02-18

**Authors:** Ivan Varga, Štefan Polák, Ján Kyselovič, David Kachlík, Ľuboš Danišovič, Martin Klein

**Affiliations:** 1 Institute of Histology and Embryology, Faculty of Medicine, Comenius University in Bratislava, 813 72 Bratislava, Slovakia; stefan.polak@fmed.uniba.sk (Š.P.); martin.klein@fmed.uniba.sk (M.K.); 2 Fifth Department of Internal Medicine, Faculty of Medicine, Comenius University in Bratislava, 813 72 Bratislava, Slovakia; kyselovic@gmail.com; 3 Institute of Anatomy, Second Faculty of Medicine, Charles University, 128 00 Prague, Czech Republic; david.kachlik@lfmotol.cuni.cz; 4 Institute of Medical Biology, Genetics and Clinical Genetics, Faculty of Medicine, Comenius University in Bratislava, 813 72 Bratislava, Slovakia; lubos.danisovic@fmed.uniba.sk

**Keywords:** telocytes, interstitial Cajal-like cells, telocytopathies, tissue regeneration, telocytoma

## Abstract

In recent years, the interstitial cells telocytes, formerly known as interstitial Cajal-like cells, have been described in almost all organs of the human body. Although telocytes were previously thought to be localized predominantly in the organs of the digestive system, as of 2018 they have also been described in the lymphoid tissue, skin, respiratory system, urinary system, meninges and the organs of the male and female genital tracts. Since the time of eminent German pathologist Rudolf Virchow, we have known that many pathological processes originate directly from cellular changes. Even though telocytes are not widely accepted by all scientists as an individual and morphologically and functionally distinct cell population, several articles regarding telocytes have already been published in such prestigious journals as Nature and Annals of the New York Academy of Sciences. The telocyte diversity extends beyond their morphology and functions, as they have a potential role in the etiopathogenesis of different diseases. The most commonly described telocyte-associated diseases (which may be best termed “telocytopathies” in the future) are summarized in this critical review. It is difficult to imagine that a single cell population could be involved in the pathogenesis of such a wide spectrum of pathological conditions as extragastrointestinal stromal tumors (“telocytomas”), liver fibrosis, preeclampsia during pregnancy, tubal infertility, heart failure and psoriasis. In any case, future functional studies of telocytes in vivo will help to understand the mechanism by which telocytes contribute to tissue homeostasis in health and disease.

## 1. Introduction

The human body consists of more than 200 morphologically and functionally different cells, although the exact number is still unclear. Additionally, most of these cells are found in different stages of their development [1]. Since the time of eminent German pathologist Rudolf Virchow (1821–1902), we have known that many pathological processes originate directly from cells. Not only did he consolidate the idea of cellular changes as the basis of disease, Virchow also advocated the promotion of histopathology to relevant and important scientific disciplines, and he riveted the attention of physicians and scientists to cells [2,3]. Perhaps Virchow’s legacy is one of the main reasons why the discovery of new cell populations within the human body is usually rooted in attempts to elucidate a disease whose etiopathogenesis is unknown. The preliminary results of such endeavors have a tendency to spark the interest of many other fellow scientists, a phenomenon known as the “snowball effect”—one study builds upon the previous one and the number of papers pointing to the importance of these cells in the pathogenesis of diseases often grows exponentially, resulting in the substantial attractiveness of the topic and quickly yielding high numbers of citations.

We hold the opinion that a similar principle also applies to a recently discovered cell population—telocytes, which have been continuously described in the pathogenesis of many different diseases over the last 10 years. Considering the extent of this publication activity, we are somewhat surprised that until now there has not been a proposal to introduce a new term referring to the group of diseases caused by the reduced number or impaired function of telocytes, for example, “telocytopathies”.

In the first place, it is necessary to point out that telocytes were discovered in 2005, only 13 years ago, in the loose connective tissue of the pancreas [4]. Because of their resemblance to enteric interstitial/pacemaker cells *(cellulae entericae interstitiales stimulantes)*, eponymously termed interstitial cells of Cajal (in recognition of the scientific contribution of the Spanish neurohistologist and Nobel Prize laureate Santiago Ramón y Cajal), the new term “interstitial Cajal-like cells” was proposed for this newly discovered population of pancreatic cells. This discovery is attributed to the team of cell biologists and pathologists from Romania led by Professor Laurentiu M. Popescu (1944–2015). Professor Popescu was considered one of the greatest Romanian cell biologists and is also renowned internationally. He was awarded multiple prestigious prizes for his scientific achievements and was selected as a president or a member of many international scientific organizations [5]. In 2000, Professor Popescu also founded the Journal of Cellular and Molecular Medicine (2017 Impact Factor 4.302). Over the following years, interstitial Cajal-like cells were described in many organs of the human body; however, it was not until 2010 when Professor Popescu, in cooperation with Professor Maria-Simonetta Faussone-Pellegrini, realized that the name “interstitial Cajal-like cells” is quite long and impractical; besides, in some organs their function was different from the original interstitial cells that Cajal found within the gut wall. The same year, Professors Popescu and Faussone-Pellegrini authored a revolutionary publication “TELOCYTES—A case of serendipity: The winding way from interstitial cells of Cajal (ICC), via interstitial Cajal-like cells (ICLC) to telocytes” [6], where they proposed the new term “telocytes” (a reference to Aristotle, according to whom “telos” was an individual’s greatest potential). As of 2018, this term has been cited, depending on the source database, more than 300 times. The discovery of telocytes was truly a lucky event since today telocytes and interstitial Cajal cells are regarded as completely different cell populations, although they can occur simultaneously in the interstitium of various organs [7]. Oddly, this revolutionary discovery of 2005 and the intense research during the following years were not enough for telocytes to be included in the official, widely accepted histological nomenclature Terminologia Histologica [8] containing all terms associated with cells, tissues and organs at the microscopic level [9]. Furthermore, telocytes are not yet mentioned in any internationally accepted textbook of histology or histopathology. This might be explained by the fact that telocytes are not widely accepted by researchers as an individual cell population. Some of them consider telocytes as only a type of “CD34-positive stromal cell”, which serve as progenitor/stem cells during the processes of regeneration and reparation [10,11,12], some think telocytes can be derived from endothelial cells [13], while others offer an alternative explanation, that telocytes could be mistaken for lymphatic endothelial cells caught in the longitudinal section [14,15]. On the other hand, several articles regarding telocytes were published in such prestigious journals *as Nature* [16] *and Annals of the New York Academy of Sciences* [17]. This could be understood as proof of at least partial acceptance of telocytes as a newly discovered and distinct cell population within the scientific community. 

The current state of knowledge indicates that telocytes form three-dimensional networks in various organs. Telocytes are typically described as cells with small bodies and one to five very long cytoplasmic processes, whose width is under the resolution power of light microscopes [18], contrasted with their length, which is considered by some authors to be the second greatest in the human body, after that of neuronal axons, reaching up to hundreds of micrometers [19]. Telocytes reportedly cover a wide spectrum of functions ranging from a role in bioelectrical activity, pacemaking and motility/contractility regulation (as within the myometrium or heart [20,21,22,23,24,25]), through the mediation of intercellular signaling between immune cells, stem cells, blood vessels or epithelial cells [26], to the orchestration of tissue regeneration and the promotion of angiogenesis, because telocytes are an important source of niche signals to stem cells [16,27,28]. Our critical review of the literature brings a concise overview of telocyte occurrence in different organs of the human body, discusses their supposed functions and last but not least ponders over the likelihood that one cell population is substantially responsible for the development of such a wide spectrum of different diseases. Thus, our principal goal is to bring a valuable contribution to the dispute of whether or not telocytes should be granted the status of an individual and unique cell population, finally puzzling out the dissonance between the myth vs. the reality attitude on the hypothesis of “telocytopathies”.

## 2. Telocytes as Principal Cells of Diverse Organs of the Human Body

In recent years, telocytes have been described in almost all organs of the human body. Although telocytes were previously thought to be localized predominantly in the organs of the digestive system, as of 2018 they have also been described in the lymphoid tissue, skin, respiratory system, urinary system, meninges and the organs of the male and female genital tracts (details in Table 1). For instance, telocytes have been detected in the stroma of the major accessory glands of the digestive system, namely in the interstitium of the pancreas [29]. They contribute to intercellular communication among pancreatic cells using gap junctions. Telocytes have also been implicated in the tumorigenesis of rare extragastrointestinal stromal tumors [30]. In the liver, telocytes have been detected in the space around the blood sinusoids [31]. Liver telocytes should be responsible for both the regeneration and fibrosis of this organ [32]. However, we could not find the essential explanation of the relationship between telocytes and stellate cells of Ito, whose role in liver regeneration and fibrosis development has been described and confirmed multiple times [33,34]. Pasternak et al. [35] used immunohistochemical methods to confirm the presence of telocytes in the muscle layer of the gallbladder. We consider this occurrence as logical, considering that the gallbladder sprouts and grows from the wall of the primitive gut, which implies that the muscle tissue of the gallbladder and the gut (the location of the interstitial cells of Cajal) has the same embryonic origin. They also revealed that the number of telocytes was lower in patients with gallstones than in healthy controls. These findings can be linked to another study in which the authors concluded that a reduction in the telocyte population could be an important etiopathogenetic factor in the development of cholelithiasis [36]. Nicolescu et al. [37] studied the salivary glands, namely the parotid gland. They demonstrated the presence of telocytes using both electron microscopy and immunohistochemistry. These techniques revealed that telocytes are located mainly in the connective tissue septa of the parotid gland, while their cytoplasmic projections connect glandular cells, blood capillaries and nerve fibers. Researchers have also been able to detect telocytes in other exocrine glands that have no developmental or other connection with the digestive system, such as the prostate [38] and mammary gland [39]. Their role in age-related degenerative changes in the prostate, characterized by alterations in the smooth muscle tone of the fibromuscular stroma, remains questionable. 

Telocytes have also been repeatedly described in the muscle tissue [40], with the bulk of attention focused on the myocardium. Interestingly, their function in the heart was shown to be considerably different from that of telocytes in other organs [41,42,43]. Recent research indicated that the subepicardial layer of the heart contains cardiogenic stem cell niches. In these specialized spots, telocytes establish a three-dimensional support network for cardiac progenitor cells. Animal-model research has shown that telocytes are present at these sites from the early stages of the heart embryogenesis, where they create a specific microenvironment, which cardiogenic cells require to properly settle and proliferate [44]. Zhou et al. [45] went even further and used the term “nurse cells”, which reflects the potential role of telocytes in the regeneration and reparation of the damaged myocardium. The role of telocytes in myocardial regeneration in humans (in contrast to animal models) remains questionable. Although telocyte numbers were shown to be reduced in end-stage failing hearts [46], in our previous study we found no link between telocyte numbers and the dynamics of the reparative mechanisms in the human heart after myocardial infarction [47]. 

## 3. Telocytes as Morphologically Unique and Functionally Diverse Cells

The morphological characteristics of telocytes have been thoroughly covered in a large number of original [83,84,85,86] as well as review papers [87,88,89,90]. The archetypal morphological signs of telocytes are small inconspicuous cell bodies [91], whose morphology is in sharp contrast with the cytoplasmic projections—telopodes [92]. These processes are some of the longest cellular processes in the human body, reaching up to several hundred micrometers in length, and their appearance is absolutely unique [93]. Telopodes are characterized as having moniliform design, with alternating thin and thick segments referred to as podomers and podoms, respectively [94]. Details regarding telocyte morphology can be found elsewhere, e.g., [95]. Typical for telocytes is also the formation of homo- and heterocellular junctions with a diverse array of cells and other tissue components, including stem cells and cells of the immune system [96,97]. Even though telocyte morphology is routinely studied with a transmission electron microscope, immunophenotyping using immunohistochemistry is also frequently employed. However, the specific immunohistochemical marker expressed only in telocytes is yet to be found. The most commonly used antibodies are those against CD34 [98], c-kit (CD117) [99] and platelet-derived growth factor receptor alpha and beta (PDGFRα and -β) [100,101] antigens. Moreover, there are also organ-specific subtypes of telocytes, which display an additional positivity, e.g., telocytes in the female reproductive system are positive for estrogen [102] and progesterone receptors [103]. Telocytes, along with fibroblasts, are currently considered constituent fixed cells of the connective tissue. Fibroblasts and their products (collagen and elastic fibers) play a role in mechano-transduction, but also importantly contribute to tissue remodeling and inflammatory processes. On the other hand, telocytes are responsible for mechano-sensitivity and contribute to reparative and regenerative mechanisms by functionally connecting different types of cells via their long cytoplasmic projections [104]. For these reasons, telocytes are frequently referred to as “connecting cells”. Telocytes are posited to have diverse roles in different organs. The most apt summary of these probable functions was provided by Creţoiu et al. [105]:

- Telocytes are cells of the connective tissue, which are functionally distinct from both stem cells and fibroblasts. Their principal functions are cellular signaling, the maintenance of tissue homeostasis and remodeling and assistance during the formation of new blood vessels (angiogenesis).
- The processes of telocytes engage in intercellular communication not only with other telocytes, but also with adjacent structures and cell populations such as blood vessels, nerve endings, smooth muscle cells and cells of glandular epithelia. This communication is enabled by homo- or heterocellular cell-to-cell junctions or by extracellular vesicles.
- Telocyte-produced extracellular vesicles play roles in the regulation of stem cell function, tissue regeneration, immunological surveillance and the maintenance of homeostatic processes.

Some researchers, like American neuroscientist Lawrence Edelstein, go even further to highlight the putative functions of this newly described cell population. Professor Edelstein compared the network comprising the processes of telocytes to a primitive cellular communication network, similar to the nervous system at the cellular level [106,107].

## 4. Diseases Associated with Telocytes, “Telocytopathies”

Telocytes were firstly linked to the interstitial/pacemaker cells of Cajal, whose detailed description served as the basis for the discovery of telocytes. The pacemaking, which controls the contractile activity of the gastrointestinal muscles, is the essence of their significant physiological role—the orchestration of the normal peristaltic activity of the digestive system [108]. The enteric interstitial cells of Cajal are also known to be the cellular origin of gastrointestinal stromal tumors (GISTs), whose characteristic histological feature is positivity for the c-kit (CD117) antigen [109,110]. GISTs are the most common mesenchymal tumors affecting the gastrointestinal tract. Interestingly, in the case of specific PDGFRα-mutant GISTs, preliminary results indicate that even telocytes can be a source cell population of this tumor [111]. Since telocytes, formerly known as interstitial Cajal-like cells, are routinely described within the glands associated with the digestive tube (the pancreas and liver with the gallbladder and hepatobiliary tree), they are implicated as the possible source of extragastrointestinal stromal tumors (eGISTs) localized in these organs [112]. Interestingly, eGISTs are rarely localized in organs outside of the digestive system, such as the prostate [113], the vagina [114], the uterus [115] or the inferior vena cava [116]. The common feature of all these tumors is the immunohistochemical positivity for the c-kit (CD117) antigen. C-kit is one of the immunohistochemical markers of telocytes in some organs, albeit an interesting observation is that specific telocytes of the gut tube are c-kit-negative [63]. Building upon the occurrence of telocytes in all of the aforementioned extragastrointestinal organs, future research will possibly reveal that eGISTs are actually “telocytomas”, tumors of telocyte origin. This term was introduced by Ricci et al. [111] in 2018. The term “telocytoma” is also applicable to other neoplasms such as inflammatory fibroid polyps usually localized in the submucosa of the gastrointestinal tract. Most inflammatory fibroid polyps bear PDGFRα-mutations (PDGFRα is one of the typical antigens of telocytes of the gastrointestinal tract), so in this case the designation “telocytoma” properly represents not only the genuine neoplastic nature of this tumor, but also its plausible histotypic lineage [111].

The diversity of telocytes extends beyond their morphology and functions, as they have a potential role in the etiopathogenesis of different diseases. The most commonly described telocyte-associated diseases (which may be best termed “telocytopathies” in the future) are summarized in Table 2. It is difficult to imagine that a single cell population could be involved in the pathogenesis of such a wide spectrum of pathological conditions. For this reason, the table should be taken with a pinch of salt, as much research remains to be done to elucidate the real importance of telocytes in these conditions. We structured the table rather unconventionally by incorporating the impact factors of the journals in which the studies were published. Our goal was to highlight the fact that even seemingly unlikely associations between these newly described cells and various acute and chronic diseases were published in prestigious scientific journals. On the other hand, a sizeable proportion of these discoveries was published in the *Journal of Cellular and Molecular Medicine*, the academic journal established by the discoverer of telocytes, Professor Laurentiu Popescu. Taking this finding into consideration may diminish the credibility of the published results regarding the supposedly groundbreaking roles of telocytes in the etiopathogenesis of various diseases. We emphasize that we are in no way casting doubt on the importance of the results published in this journal. 

In the recent literature, few human studies have investigated changes in the distribution and function of telocytes during different diseases; most studies of this kind have used animal models. Moreover, the causative relationship between a quantitative reduction of telocytes and disease development is disputable. It is important to consider that the modification of the cellular microenvironment of different organs during a disease can be the cause of telocyte loss, rather than the other way around.

## 5. Conclusions

Resolving the status of telocytes as an individual cell population will be important for morphologists, who still hesitate to include telocytes in histology and cellular biology textbooks and to incorporate them into the histological nomenclature. Tissue engineers are also interested in the final answer regarding the role of telocytes as stem cell “nurses” and their importance in regenerative medicine. Telocytes as “nurse” cells were reported, for instance, in the intestinal epithelium [16] and within the myocardium [137]; the discovery of new players in the regeneration and reparation of these organs would be revolutionary for future therapeutic applications. For pathologists and clinicians, the real role of telocytes in the pathogenesis of diseases with different etiologies (“telocytopathies” and “telocytomas”) remains unresolved. The most interesting aspect for immunologists is that a portion of the telocyte population may participate in the education of cells of the immune system [138]. In any case, future functional studies of telocytes in vivo will help to understand the mechanism by which telocytes contribute to tissue homeostasis in health and disease.

## Figures and Tables

**Table 1:** Typical locations of telocytes in mammals (organs are in alphabetical order).

Organ or Tissue	References
Blood vessels	Cantarero et al. [48]
Bone marrow	Li et al. [49]
Eye (sclera, limbus, uvea and lamina fusca)	Luesma et al. [50]; Petrea et al. [51]
Fascia lata	Dawidowicz et al. [52]
Heart, including endocardium, myocardium, epicardium and heart valves	Gherghiceanu et al. [53]; Kostin [54]; Popescu et al. [55]; Yang et al. [56]
Kidney	Qi et al. [57]; Rusu et al. [58]
Liver and gallbladder	Xiao et al. [31]; Matyja et al. [59]
Mammary gland	Gherghiceanu and Popescu [39]
Meninges and choroid plexus	Popescu et al. [60]
Neuromuscular spindles	Díaz-Flores et al. [61]
Esophagus and stomach	Rusu et al. [62]; Vannucchi et al. [63]
Pancreas, exocrine part	Nicolescu and Popescu [29]
Placenta, placental chorionic villi	Bosco et al. [64]; Nizyaeva et al. [65]
Prostate	Shafik et al. [38]; Corradi et al. [66]
Salivary glands	Nicolescu et al. [37]
Skeletal muscle (within its interstitium)	Marini et al. [67]
Skin	Rusu et al. [68]
Small and large intestines	Cretoiu et al. [69]; Vannucchi et al. [63]
Spleen	Chang et al. [70]
Synovial membrane	Rosa et al. [71]
Temporomandibular joint disc	Rusu et al. [72]
Trachea, lungs and pleura	Rusu et al. [73]; Popescu et al. [74]; Hinescu et al. [75]
Trigeminal nerve ganglia	Rusu et al. [76]
Urinary bladder and ureters	Vannucchi et al. [77]; Rusu et al. [78]
Uterine tubes and uterus, including endometrium, myometrium and cervix	Popescu et al. [17]; Cretoiu 2016 [79]; Hatta et al. 2012 [80]; Creţoiu et al. [19]; Klein et al. [81]
Vagina	Shafik et al. [82]

**Table 2:** Summary of diseases associated with a reduced number or functional disorder of telocytes.

Organ System	Organ, Tissue	Disease, “Telocytopathy”	Authors and Year of Publication	Journal Name and Impact Factor
Digestive system	Gallbladder	Gallstones	Matyja et al. [59]	*J Cell Mol Med* 3.698
	Liver	Liver fibrosis	Fu et al. [117]	*J Cell Mol Med* 4.938
	Pancreas	Extragastrointestinal stromal tumor	Padhi et al. [30]	*JOP* 0.0
	Gut	Ulcerative colitis	Manetti et al. [118]	*J Cell Mol Med* 4.938
	Gut	Crohn’s disease	Wang et al. [119]	*Neurogastroenterol Motil* 3.364
	Salivary glands	Sjögren’s disease	Alunno et al. [120]	*J Cell Mol Med* 4.938
	Gastric antrum	Inflammatory fibroid polyp	Ricci et al. [111]	*J Cell Mol Med* 4.302
Respiratory system	Lungs	Fibrosis after pneumonia	Sun et al. [121]	*J Cell Mol Med* 4.014
	Larynx	Reinke’s edema	Díaz-Flores et al. [11]	*Semin Cell Dev Biol* 6.614
Urinary system	Kidney	Ureteropelvic junction obstruction	Mehrazma et al. [122]	*Iran J Pediatr* 0.522
	Urinary bladder	Neurogenic detrusor overactivity	Gevaert et al. [123]	*J Cell Mol Med* 4.125
Male genital system	Testes	Hyperplasia of Leydig cells in undescended testes (cryptorchidism)	Díaz-Flores et al. [11]	*Semin Cell Dev Biol* 6.614
	Prostate	Prostate cancer, benign prostate hyperplasia	Gevaert et al. [124]	*Histopathology* 3.453
Female genital system	Placenta	Preeclampsia	Bosco et al. [64]	*Med Hypotheses* 1.136
	Uterine tube	Endometriosis, uterine tube damage, infertility	Yang et al. [125]	*J Cell Mol Med* 4.938
	Ovaries	Premature ovarian failure	Liu et al. [126]	*Mol Med Rep* 1.692
	Uterus	Uterine fibroids (leiomyomas)	Varga et al. [127]	*Med Hypotheses* 1.120
Skin and skin derivatives	Skin	Psoriasis	Manole et al. [128]	*J Cell Mol Med* 4.938
	Skin	Basal cell carcinoma, squamous cell carcinoma	Mirancea et al. [129]	*Rom J Morphol Embryol* 0.723
	Mammary gland	Breast cancer	Mou et al. [130]	*J Cell Mol Med* 3.698
	Skin and internal organs	Systemic sclerosis	Manetti et al. [131]	*J Cell Mol Med* 4.014
Sensory system	Eye	Keratoconus	Marini et al. [132]	*J Cell Mol Med* 4.302
Cardiovascular system	Heart	Heart failure	Richter and Kostin [46]	*J Cell Mol Med* 4.938
	Heart	Heart attack	Galrinho et al. [133]	*Maedica (Buchar)* 0.0
	Heart	Arrhythmia	Hinescu et al. [134]	*J Cell Mol Med* 6.555
	Blood vessels	Vascular hyperplastic diseases	Li et al. [135]	*Sci China Life Sci* 2.781
Connective tissue	Fascia lata	Various degenerative changes	Szotek et al. [136]	*Ultrastruct Pathol* 0.694
